# Amorphous Polymers’ Foaming and Blends with Organic Foaming-Aid Structured Additives in Supercritical CO_2_, a Way to Fabricate Porous Polymers from Macro to Nano Porosities in Batch or Continuous Processes

**DOI:** 10.3390/molecules25225320

**Published:** 2020-11-14

**Authors:** Margaux Haurat, Michel Dumon

**Affiliations:** Laboratoire de Chimie des Polymères Organiques (LCPO), UMR 5629, Bordeaux INP/ENSCBP, University Bordeaux, CNRS, 16 Avenue Pey-Berland, CEDEX, F-33607 Pessac, France

**Keywords:** polymer foam, nanostructuration, batch-foaming, PMMA, MAM, core-shell particles, supercritical CO_2_, foaming processes

## Abstract

Organic polymers can be made porous via continuous or discontinuous expansion processes in scCO_2_. The resulting foams properties are controlled by the interplay of three groups of parameters: (i) Chemical, (ii) physico-chemical, and (iii) technological/process that are explained in this paper. The advantages and drawbacks of continuous (extrusion, injection foaming) or discontinuous (batch foaming) foaming processes in scCO_2_, will be discussed in this article; especially for micro or nano cellular polymers. Indeed, a challenge is to reduce both specific mass (e.g., ρ < 100 kg·m^−3^) and cell size (e.g., average pore diameter ϕ_average_^pores^ < 100 nm). Then a particular system where small “objects” (coreshells CS, block copolymer MAM) are perfectly dispersed at a micrometric to nanometric scale in poly(methyl methacrylate) (PMMA) will be presented. Such “additives”, considered as foaming aids, are aimed at “regulating” the foaming and lowering the pore size and/or density of PMMA based foams. Differences between these additives will be shown. Finally, in a PMMA/20 wt% MAM blend, via a quasi one-step batch foaming, a “porous to nonporous” transition is observed in thick samples. A lower limit of pore size (around 50 nm) seems to arise.

## 1. Introduction

In the huge field of porous materials, porous organic polymers have been elaborated for a long time by a lot of methods [1,2,3], involving either chemistry [4,5] or physical means [6,7]. These materials are also named cellular, lightweight materials, sieve-like, membrane-like, sponges, or foams.

On the side of processes, physical foaming is one method where a volume expansion takes place in the polymer, through a gas depressurization (CO_2_, N_2_, etc). This method has the advantage of a fast process time, a relative low cost, it is applicable to several polymers and is an alternative to the classical chemical foaming (CBA)—some of which tend to be forbidden by REACH regulation (such as azodicarbonamide).

On the side of pore structure, an ultimate goal is to reduce both specific mass (e.g., ρ < 100 kg·m^−3^) and cell size (e.g., average pore diameter ϕ_average_^pores^ <100 nm). These features are hardly achievable when a single polymer is used. A literature survey of the last ten years reveals some works and methods on cellular bulk micro [8,9,10,11,12] and nano [13,14,15,16,17] polymer foams in a cellular size range around or just below one micron.

Nano cellular polymers (voids < 0.1 µm) is a rather new class of foams that can be found, so far, mainly in polymer formulations, in structured polymers, in polymer blends or in nano composites [5,14,18,19,20]. Furthermore, most of the time, they are obtained in batch, i.e., a discontinuous process; as shown in the literature since 2002 [21,22,23,24,25]. The industrial (and even scientific) challenge is now to produce this kind of foams following a continuous process (such as extrusion foaming) [26,27,28].

Generally speaking, the pore-generation methods are very numerous, ranging from solvent phase separation—evaporation, degradation, extraction, leaching, and gas foaming methods. All methods cannot be discussed extensively so we will concentrate on supercritical fluid assisted foaming (scCO_2_ in this article) [29].

Among foaming methods, the gas introduction is either direct—the method is named “physical foaming”; or by blending with a gas-generating molecule (CBA)—the method is named “chemical foaming”. Three types of chemical and physical data are ruling the foam formation: (i) Pressure and temperature [30,31], (ii) capacity of the polymer to deform (melt strength), (iii) chemical affinity (solubility, swelling) of the polymer in the gas and the fact that the gas moves more or less easily in and out from the polymer (diffusion, permeability) [32].

Usually, swelling and solubility (being the two responses of a polymer to CO_2_ sorption) are correlated but they do not necessarily vary with the same magnitude, i.e., a polymer may have a small swelling and a high solubility.

Upon decrease of pressure or temperature, a one-phase (gas-polymer) system becomes a two-phase system, i.e., a foam composed of gas cells (CO_2_, N_2_ or air) + polymer walls or struts. When scCO_2_ is used as a blowing agent, a very difficult objective is to master the expansion at a very small size (nano) while having a low density. In a continuous process, the problems for keeping the foams at a nano level are indeed not yet solved [26]. Thus, nano and even micro gas-foamed bulk polymers still remain a challenge for continuous production.

Microcellular CO_2_-foamed polymers are characterized by a cell density greater than 10^10^–10^9^ cells·cm^−3^ and a cell size in the order of 1 to 10 μm. Yet cases for microcellular polyolefins and polystyrene have been cited [33,34,35]. At an effective large fabrication scale, the MuCell process is nearly the only process operating for injection molded micro foams (https://trexel.com/technology-solutions/mucell), with some other less known methods [36,37].

Wrongly speaking but often used for polymer foams, the word “nano” is considered if the mean pore size is less than about 80 nm; which is different from the IUPAC terminology: Microporous 0.2–2 nm, mesoporous 2–50 nm, macroporous > 50 nm.

In the domain of sub micrometric porous materials (e.g., pores < 1 µm), several methods are well known such as “templating or templates”, “emulsion(s)”, “sacrificial methods”, “sol-gel methods”, “phase separation” methods, and “solvent methods”, which are most of the time discontinuous and non-foaming processes. Although allowing very small pore sizes (<<50 nm), these previous methods are not really challengers to foaming. They are used for small thickness pieces (e ≤ 1 mm), in thin layers or films but never for continuous bulk monoliths.

In polymers, the range of sub micrometric pores is expected to exhibit “high” physical properties. Indeed, several physical properties exhibit a step evolution in the porosity window from “micro” to “nano” (typically below 80 nm). Such evolutions are expected for mechanical damping, specific absorbed energy, rigidity, acoustic properties, electromagnetic sheltering, filtration or separation and most of all thermal conductivity [2,17,38,39,40]. Moreover, transparency would be a further advantage. For comparison, mainly inorganic, based on silica, or silica/organic polymer hybrids, (seldom neat polymer aerogels) provide extremely light bulk nanometric or submicronic porous materials (both semi-transparent and insulating). Transparency in amorphous polymer nano foams was cited as a possible property by Dumon et al. [10]; it was developed theoretically by Perez Tamarit et al. [41], then studied and proved on PMMA by Martin de Leon et al. [42,43,44]. The previous authors showed that combination of rather difficult conditions (low saturation temperatures e.g., −32 °C) with high saturation pressures (e.g., 20 MPa), i.e., high CO_2_ solubility and high nucleation density) are needed to reveal very good transparency (with sample thickness much less than 1 mm) or semi transparency (with sample initial thickness of 2 mm). The fabricated homogeneous PMMA foams have cells all below 50 nm and density around 500 kg·m^−3^. They also proposed models for scattering and effect of wave lengths.

Inorganic aerogels (semi-transparent, and extremely light) have, so far, the lowest known values of thermal conductivity, λ^therm^ down to 15 mW·m^−1^·K^−1^. Thus, nano porous polymers are for example expected to be useful for super insulation thermal applications [40,45,46] with better mechanical resistance than inorganic aerogels. Yet, if inorganic aerogels are constantly improving to solve the antagonism between a poor mechanical behavior and very high thermal performances, they still need a long synthesis process (multi steps, expensive products), with fragile textures; they remain sensitive to wear, to friability and dusting.

The aim of this article is to analyze how micro and nano cellular foams from amorphous polymers are prepared and controlled through a supercritical fluid saturation (mainly CO_2_) followed by an expansion step, via different processes. Furthermore, the paper provides comparative chemical physico-chemical literature data. Then we will give results on a specific polymer (PMMA, poly(methyl methacrylate)) blended with CO_2_-philic additives (core shell particles CS) or nano structured acrylic block copolymers, named MAM [47,48]. Acrylic core shell particles are chosen in the Durastrength^®^ range, with either a crosslinked core or a liquid core, used classically as impact modifiers. MAM belong to the range of poly(methyl methacrylate-co-butylacrylate-co-methyl methacrylate) block copolymers—Nanostrength^®^ range.

## 2. Results and Discussion

### 2.1. Analysis of Literature Results: scCO_2_ Foaming Processes in Organic Polymers: Parameters Influencing Foaming, Batch vs. Continuous

CO_2_ is the principal molecule to provide an easy supercritical state (roughly above 35 °C, 7.5 MPa) and is chemically unreactive to most polymers. Even if N_2_, H_2_O and gas mixtures are also used in the supercritical state, most of the physical foaming processes are done with CO_2_.

scCO_2_ is not rigorously speaking a blowing agent such as CBA—chemical blowing agents, that are molecules decomposing chemically upon heating, releasing gas molecules (CO_2_, water, nitrogen, etc.); but scCO_2_ is called a physical blowing agent. Although scCO_2_ has advantages, e.g., it is considered as a “green”, non-toxic, and low cost molecule, it has a rather low solubility (Table 1) and a rather slow diffusivity in organic polymers.

Polymer foaming results from an interplay of three groups of parameters: (i) Chemical, (ii) physiso-chemical, and (iii) technological/process.

#### 2.1.1. Chemical Parameters

They relate to macromolecules chemical composition, chain length (molar mass) and their CO_2_ solubility.

CO_2_ solubility (%), also named CO_2_ uptake or CO_2_ mass gain or CO_2_ sorption, is defined as the ratio of mass gain of CO_2_ after an equilibrium saturation step at a given temperature and pressure (Equation (1)). In literature [49], solubility is expressed in the either of the following units: % CO_2_ uptake, or mass of CO_2_ in 1 g of polymer after saturation (P,T), or mol^gas^/kg^polym^ (=0.227 × % CO_2_ uptake) or cm^3^ (STP)gas/cm^3 polymer^ (we made the reasonable approximation that % CO_2_ uptake non STP (i.e., RT) ~ 0.1626 × V^CO^_2_ (STP)). Table 1 gives a comparative list of solubility in different polymers where values have been translated in the same unit (% CO_2_ uptake, i.e., mass of CO_2_ in 100 g polymer).

(1) $${\%\mathrm{CO}}_{2}\mathrm{uptake}=100\times \frac{m_{{\mathrm{CO}}_{2}\;\mathrm{saturated}\;\mathrm{sample}}-m_{\mathrm{non}\;\mathrm{saturated}\;\mathrm{sample}}}{m_{\mathrm{non}\;\mathrm{saturated}\;\mathrm{sample}}}$$

While CO_2_ is a good solvent for many non-polar (and some polar) low molar mass molecules, it is a poor solvent for macromolecules under readily achievable conditions (e.g., 100 °C, 10 MPa).

Thus, CO_2_ has a “moderate capacity” to expand polymers, although it is definitely used because the supercritical conditions offer advantages such as a liquid-like solubility and an enhanced diffusivity.

Table 1 shows that the only polymers that have good solubility in CO_2_ under mild conditions are certain amorphous fluoropolymers (e.g., poly(perfluorooctyl acrylate) PFA/PPFA) and silicones (PDMS—poly(dimethyl siloxane)). Generally, all amorphous fluorinated copolymers are stated to be very soluble in scCO_2_ [77,78] but exact % CO_2_ uptake values are not reported in literature; solubility is assumed via phase diagrams or structure/relationships studies [77,78].

The relative high solubility of amorphous fluoropolymers may be explained by weak complexes with CO_2_, or by preferential clustering of CO_2_ near the fluorine atom of the C-F bonds, which are more polar than C-H bonds. Hence, fluorinated side groups may “shield” the hydrocarbon main chain from interacting with the solvent [79]. Li et al. [80] have shown that it is possible to enhance the CO_2_-solubility of a fluorinated polymer foam (poly(perfluorooctylethyl methacrylate): PFMA) by reducing its depressurization temperature from 0 to −40 °C.

The solubility of silicon polymers (e.g., PDMS) is enhanced by the very flexible nature of these chains that provides them with large free volume (PDMS have the lowest T_g_ among polymers).

The presence of carbonyl functions, in polyesters or polyacrylates (e.g., PMMA), tends to increase CO_2_ solubility. This is the reason why we will use PMMA as a model system.

To further increase CO_2_ uptake, low molar mass CO_2_-philic additives can be added. On the contrary, CO_2_ loss is favored by the existence of sharp immiscible interfaces, for example when CO_2_-unsoluble fillers are added. Expecting a high CO_2_ uptake, CS and MAM additives are chosen since their acrylic nature implies no sharp interfaces in PMMA [81].

In the molten state, i.e., at high temperatures (typically above 160 °C), under high pressure (typically above 10 MPa), a polymer/gas one-phase solution is observed. On the one side, gas sorption causes the polymer solution to swell; on the other side, a high hydrostatic pressure causes the polymer chains to “contract” under stress, i.e., to pack, or modify their entanglement.

#### 2.1.2. Physico-Chemical Parameters

They relate to phase structure (e.g., crystalline areas, areas with a nano structuration as micelles, core shell particles, and lamellas) and to viscoelastic behavior (chain mobility, viscosity, flow and stretchability) [46,82]. Semi crystalline polymers (mainly PE, PP), although “CO_2_-foamable”, constitute a separate case due the impermeability of the crystalline or organized areas [83,84]. Our work is discussing only amorphous polymers and their blends for the foaming process and technology.

The expansion ratio varies with the polymer state: It is limited in glassy solid, easier in viscoelastic solid (solid state foaming), and much easier in melt foaming [85]; but consequently, porous morphologies are more difficult to stabilize due to the chain mobility increase.

Expansion (closed or open cells) is triggered by a gas depressurization, after saturation in supercritical conditions (scCO_2_, scN_2_). Depressurization induces a phase separation from a one-phase gas-saturated polymer/gas system to a two-phase polymer/pore system [86]. This phenomenon has been observed through a sapphire window during a PP batch-foaming in scCO_2_ (10 MPa, 180 °C during 30 min). It is visually observable with bubbles apparition in the sample that was homogeneous before depressurization [87]. At the moment of foaming (a fraction of seconds to some minutes), the “gas foaming molecules” are in a “true” gaseous state (not supercritical); while those molecules are in the supercritical state during the saturation period (several minutes to several days depending on the process).

Gas solubility, diffusivity and pressure drop rates are first considered as the ruling parameters, then technological parameters such as choice of process (batch vs. continuous, equipment, tooling, time) come into account. To give an order of times involved, in batch-foaming: CO_2_-saturation is on the order of hours (solid state bulk pieces, >2 mm), one-step foaming is on the order of one minute and two-step foaming is on the order of some minutes [58]. In the melt state, the underlying scientific problem is the coupling of rheology, thermodynamics and the sorption/desorption/diffusion kinetics, and final foaming, in a short time (1 to 4 min) [1].

#### 2.1.3. Technological/Process Parameters

**Figure 1:**
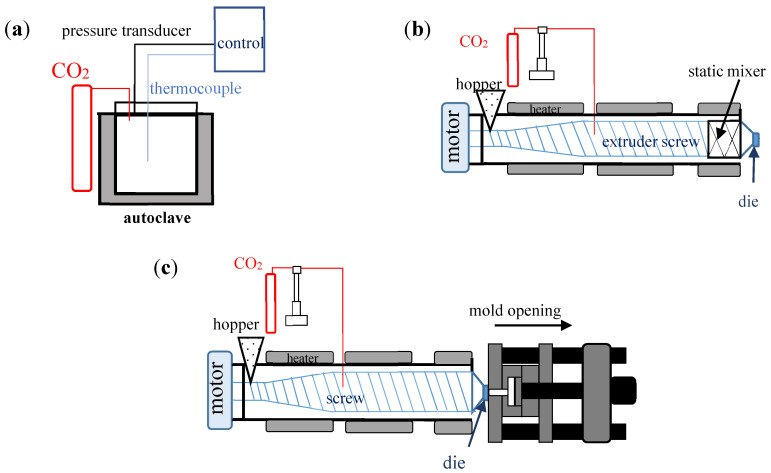
Different foaming processes using supercritical fluids as blowing agents: (**a**) Batch-foaming, (**b**) extrusion foaming, (**c**) injection foaming.

They relate to pressure, temperature, tooling, and equipment. Different processes can be followed to produce solid foam in presence of a supercritical fluid: Batch-foaming, extrusion-foaming or injection foaming (Figure 1). For each one, it is possible to modify several technological parameters in order to improve foaming.

No matter the process, the blending ability is first crucial to ensure the homogeneity of the blend, the diffusivity and the solubility of the gas into the material.

In the case of the extrusion process, scCO_2_ is nevertheless an extrusion aid by acting as a plasticizer (chains disentanglement, increasing chain mobility); glass transition temperature (T_g_) and viscosity (η) of the CO_2_/polymer mixture are greatly decreased [88].

Various parameters, such as pressure, temperature and saturation time, are adapted considering the process (batch, extrusion or injection foaming). Indeed, they will have a specific impact on the foaming depending on the process (Table 2).

In batch foaming, pressure is easily controllable, whereas in extrusion foaming it is directly linked to the extrusion rate and the screw speed [93]. In injection molding, pressure is controlled as in extrusion foaming but the mold pressure also impacts the foaming [94]. For each process, pressure–temperature parameters can be considered as pair parameters because the pressure applied varies with temperature. So, the technological parameters that influence the temperature also impact the pressure.

As indicated in Table 2, it is harder to control saturation and foaming pressures–temperatures in extrusion and injection than in batch.

For example, in extrusion, temperature adjustment is applied all along the barrel up to the die. If the die temperature increases, the diffusivity of the gas will increase while the viscosity of the polymer will be lowered. In these conditions, the cell walls will not be rigid enough to keep the CO_2_ in the foam at the beginning of foaming. Expansion may be easy at first, but walls shrink, and final expansion will be low. If the temperature is too low, the polymer becomes too rigid, becomes non extrudable, and the expansion is limited [95].

A minimum (critical) saturation time is crucial in all the processes, it determines the CO_2_ amount that can be added into the material. In batch foaming this time is independent of the technological parameters but this is not the case for the two others processes where it is linked to the screw speed for extrusion foaming [93] and the molding cycle chosen for injection foaming. To reach smaller cells in injection foaming it is possible to adjust the dwelling time. Increasing this time, the polymer melt strength will increase what will lead to a restricted cell growth and a reduction of coalescence phenomena [36].

In extrusion foaming it is nevertheless possible to partially control saturation time by adding elements, such as a static mixer, at the end of the extruder [96]. This kind of modification can also be a way to ensure the homogeneity of the blend and to reduce its temperature before depressurization.

Finally the geometry of the die (capillary, flat die, bent die, etc.) [93], and its diameter for capillary dies, directly bias the cell density and the expansion of the foam. Depressurization happens at the end of this zone when the material goes out and passes through the die to ambient pressure. For capillary dies, pressure drop rate increases when the die diameter decreases and causes an increase in the nucleation rate that leads to a density reduction, as shown for example in stark based materials [97] and polystyrene (PS) [98]. For bent dies, there are some more pressure losses due to the energy dissipation by friction; these losses can stabilize the polymer flow and thus facilitate the control of the expansion at the end of the die [98].

### 2.2. New Examples Based on Amorphous Polymer (PMMA) Batch Foaming: PMMA Blends with Core–Shell Performed Particles (CS) or a Structured Acrylic Block Copolymer (MAM), as CO_2_-Philic Foaming-Aid Additives

This paragraph gives specific results on PMMA blends with 20 wt% of a CO_2_-philic structured additive (MAM or CS) dispersed as “very small objects” in the PMMA matrix. The result is a structured blend where the compatibility between phases is excellent (no sharp interfaces); these additives are considered as “foaming-aids”, especially to the nano or micro range.

MAM, i.e., PMMA-co-PBA-co-PMMA block copolymer, is organizing as micellar objects at 20 wt% in PMMA and used for a foaming aid in PMMA foams [9,10,99]. Core shells (from Durastrength series) are preformed acrylic spherical particles easily dispersible in PMMA and not yet used in polymer foams.

In batch foaming process, polymer samples are saturated in a scCO_2_ vessel (also named reactor) at T^saturation^ (typically between RT and 80 °C; it can be lowered down to 0 °C to increase CO_2_ solubility [42,80,100]) and P^saturation^ (typically 5 to 30 MPa) for a time, t^saturation^, (typically 12 h to several days). After saturation, the solid material is back to RT and atmospheric pressure.

Depending on the state of the polymer at the moment of expansion: Glassy solid or rubbery solid. Indeed, the value of Tg of the polymer/CO_2_ mixture depends on P^saturation^. Indeed, the value of Tg of the polymer/CO_2_ mixture depends on P^saturation^. CO_2_ acts as a plasticizer so that Tg of the polymer/CO_2_ mixture is always lower than that of the polymer (20 to 50 °C lower). This plasticization enables batch one-step foaming. Heating in the two-step-foaming process gives mobility to the chains and allows expansion at a temperature above Tg of the polymer/CO_2_ mixture at atmospheric pressure.

MAM is known to regulate (to homogenize) cell size distribution and tends generally to lower the cell size of batch PMMA foams. PMMA/MAM being a good model system, a huge collection of experiments on batched-foamed PMMA/MAM structured blends was published varying wt% MAM (0.1 to 20 wt%), P^saturation^, T^saturation^, ΔP/dt, one-step vs. two-step foaming, T^foaming^. All works show that density is accessible between 1 and 0.25·10^3^ kg·m^−3^, mean pore diameter between several tens of micrometers to 0.1 µm, exceptionally down to 50 nm (thanks to hard low temperature saturation conditions) [39,81,89,101,102].

The PBA rubbery block (very low T_g_, never vitrifying) has a higher CO_2_-affinity than the less CO_2_-philic PMMA side blocks which are able to vitrify, T_g_ being around 110 °C. Thanks to this PBA block, under the same saturation conditions (e.g., RT, 30 MPa during 16 h), MAM has a higher CO_2_-philicity (e.g., CO_2_ uptake: 24.1 wt%) than PMMA (e.g., CO_2_ uptake: 12.1 wt%) [12].

Finally, MAM micelles act as CO_2_ reservoirs during the saturation and due to the CO_2_ amount added into the material, the cell density will be increased whereas growth and coalescence would be limited below T_g_.

Here we investigate the role of two additives vs. type of foaming and their relative or mutual influence. We use two “foaming aids” and submit them to different types of foaming. In Table 3 and Table 4 two sets of experiments (I, II) are presented in PMMA-based foams.

**Figure 2:**
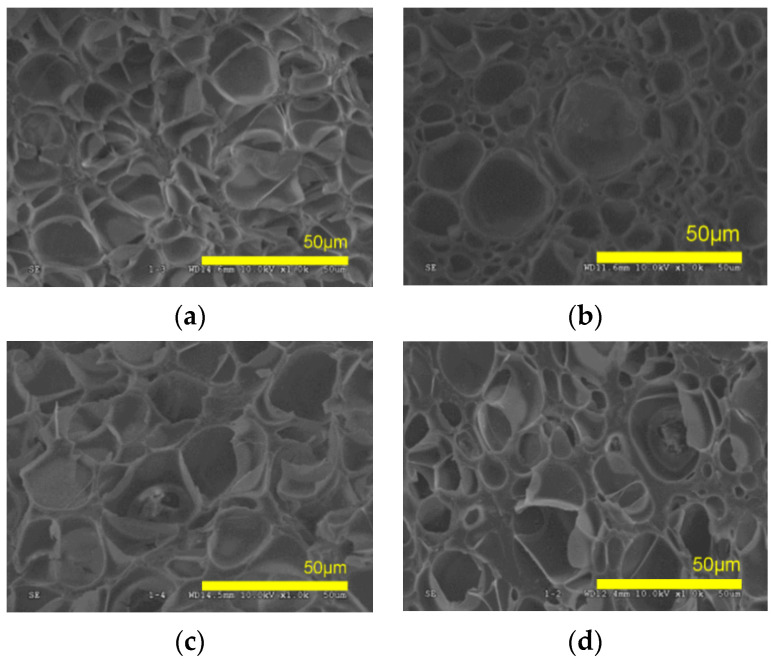
SEM microstructure of poly(methyl methacrylate) (PMMA)/20 wt% additive based foams in Set I (**a**) D200, (**b**) D480, (**c**) Dlab, (**d**) block copolymer MAM.

Set “I” (Table 3) compares the use of the two additives (MAM, CS) at 20 wt%, after a one-step batch foaming (saturation at 80 °C, 31.5 MPa, ΔP/dt ~12 MPa·min^−1^). This choice is always inducing expansion upon depressurization. Foams’ morphologies are shown on SEM (Figure 2). The average pore diameter classically lies in the micrometer range in one-step batch foams from a temperature where the system is rubbery and expandable.

Set “II” (Table 4) uses of a quasi-one-step batch foaming, i.e., samples are left either at RT after depressurization **or** immersed in ice bath (IB) just after depressurization. According to saturation temperature and pressure, after the pressure drop, this method induces or not an expansion. The expansion is so qualitatively observed with time. A gradient of opacity may grow through a sample, from transparent state at the end of depressurization.

#### 2.2.1. Comparison of CoreShell and MAM Tri Block Copolymer

For set I, saturation at 80 °C, 31.5 MPa (Table 3), no real difference appears in density (ρ) between the two crosslinked-core CS and MAM. But there is a noticeable lower density for the liquid-core CS (0.24·10^3^ kg·m^−3^), in accordance with its better capacity to swell, particularly for foaming at RT. However, the average diameters are comparable for all CS’s and MAM, between 12.5 and 15.5 µm.

**Figure 3:**
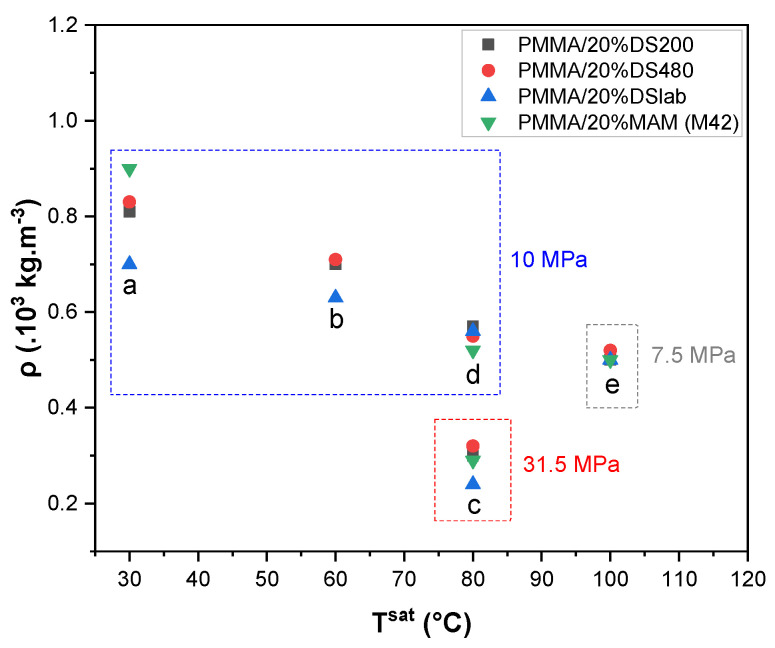
Evolution of foams density, one-step batch-foamed with scCO_2_ various saturation temperature and pressure. Pressure is indicated with a frame and each group of points is marked with a letter also shown in Table 4.

For the set of experiments II, the data of Table 4 are plotted in Figure 3, where ρ = f(T^saturation^). Set II is a quasi-one-step batch foaming from different saturation temperatures (T^foaming^) and pressures (P^saturation^). CS’s and MAM behave rather similarly; each additive lies close to each other for a pair of (P^sat^, T^sat^). In these experiments, one observes that T^sat^ and P^sat^ are the influencing parameters. The classical dependence of decreasing density with T^sat^ and P^sat^, independently of the additive, are shown in Figure 3. Indeed at 10 MPa, density decreases linearly (from ~0.8·10^3^ to ~0.5·10^3^ kg·m^−3^, while the temperature increases (from 30 °C to 80 °C). On another side, at a given temperature (80 °C) the density decreases while the pressure increases.

Interestingly, things are somehow different at low saturation temperatures (e.g., ≤30 °C). Even more in ice, the role of vitrification prevails and stops expansion for all systems (no foaming, denoted as NF). This role is attributed to vitrification of both PMMA matrix, shells or side PMMA blocks, and the fact that T_g_ of CO_2_-swollen PMMA lies above 0 °C. Thus it appears that liquid-core CS have in interest in low temperature foaming.

#### 2.2.2. Role of a Quasi One-Step Batch Foaming

The occurrence of gradient or gradient-like porosity or a sharp nonporous/porous transition at a porosity scale of tens of nm on thick samples was investigated. Thick samples have an intrinsic thermal insulation that enables temperature gradients during foaming, and therefore the presence of potential differently foamed areas, or layers in which foaming would be changed layer by layer. We also want to see if pore size is reduced (or not) in the different potential foaming areas. So blend samples of PMMA/foaming-aid nano (MAM) or micro (CS) structured additives are submitted to batch foaming in view of creating inhomogeneous foams, for example true gradient foams or either a material with a sharp porous/non porous transition. For this, foaming needs to be quickly limited by vitrification or by CO_2_ diffusion. Note that in batch solid-state-foaming, gradient effects are possible only in thick insulating samples and thanks to a post temperature effect.

To do so, we focused on the effect of a simple post temperature treatment. Samples are treated in by a quasi one-step batch foaming, i.e., classically saturated in a pressure vessel, depressurized at room temperature (foamed or not, depending on conditions) and immersed immediately in an ice bath (instead of remaining at RT).

We characterized samples both by visual and SEM observations. First porosity is not present over the whole sample. Then the level to which pore size may be reduced is looked for, while maintaining an expansion (i.e., a density reduction).

**Figure 4:**
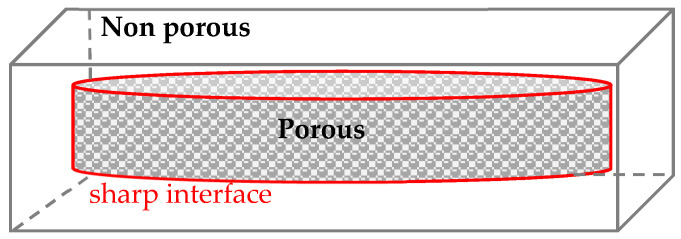
Scheme of a bulk sample of PMMA/20 wt% additive (D200, D480, Dlab or MAM) foamed following Set II.

**Figure 5:**
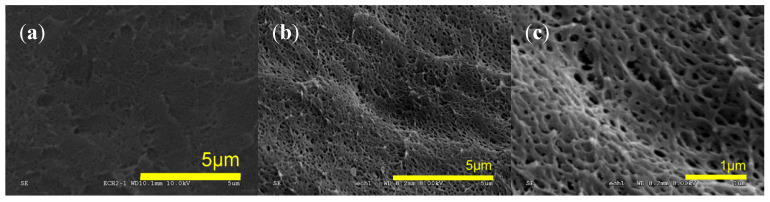
Set II PMMA/20 wt% MAM ice immersed after batch foaming at 10 MPa and 30 °C, (**a**) area of the sample where a transition transparent to translucent is observed, that is a “nonporous to porous” transition (reminder: Sample is thick), (**b**,**c**) area of the sample where opacity (even slight) is observed, that is a porous region (reminder: Sample is thick).

Figure 4 depicts a scheme of a sample exhibiting an area where a transition from translucent to opaque appearance is observed. Nanopores are present and rather well distributed in the observed opaque areas. SEM micrographs (Figure 5a) show only a few nano cells in the range of 50 nm in a translucent area (unfoamed). But in the porous areas (Figure 5b,c), a lot of nano cells are indeed well distributed with cell size between 50 to 120 nm. However, SEM reveals that the transition from dense to porous is sharp. This porosity results from a temperature gradient through the insulating character of thick samples during foaming and from a gradient of CO_2_ diffusion (the central area is hotter and richer in CO_2_, it can foam).

Thus, through this experiment, we show that a true porosity gradient is not revealed; and there seems to be a porosity “discontinuous” transition in batch foamed thick samples (minimum 2 mm thick). It is also shown in our conditions that pore size lower than 50 nm cannot be generated.

## 3. Experimental Section

### 3.1. Materials and Unfoamed Precursors Production

Details on initial materials (PMMA, MAM, CS) can be found in the literature [20,47,48]. All of them were kindly supplied by Arkema (Lacq and Lyon, France).

Materials, as pellets, were first dried at 80 °C during 4 h before processing. Then PMMA/20 wt% additive blends were compounded using a Scamex CE02 (Scamex, Crosne, France) single-screw extruder (L/D = 28; d = 45 mm), with a temperature profile from 165 to 225 °C, at a screw speed of 60 rpm. Pellets were produced using a continuous cutting machine operating at the end of the line. Then, the pellets were dried again (8 h at 80 °C) before being injected as tensile test bars (ISO 180/U 80 × 10 × 4 mm^3^) by a classical injection molding (DK 50T, DK Technologie, Gonesse, France), with a screw temperature of 240 °C and a mold temperature of 50 °C. The injected samples present the aspect and the properties shown in Table 5.

### 3.2. Porous Samples Production

All the materials presented in the article were foamed in batch foaming in presence of supercritical carbon dioxide (scCO_2_) at 99.9% pure from Air Liquide (Grand Couronne, France). The experiments were done into a high-pressure vessel provided by TOP Industrie (Vaux-le-Pénil, France). This vessel has a capacity of 300 cm^3^ and it is possible to use it up to 40 MPa and 250 °C. The pressure and temperature are kept at desired values through a pressure pump controller Teledyne ISCO model 260 (Teledyne ISCO, Lincoln, USA).

In this study the samples were saturated at different pressures and temperatures during 24 h to ensure the CO_2_ dissolution in the polymer. Two kinds of experiments were conducted.

The first one, called Set I, corresponds to a “one-step” batch-foaming method. The samples (PMMA/20 wt% MAM and PMMA/20 wt% CS) are saturated at 80 °C and 31.5 MPa during 24 h before the depressurization. These conditions have been selected to be sure that the samples always expand after depressurization. When the pressure is released, the temperature shows a great drop [9,10] and we go from supercritical conditions to ambient conditions.

The second method, called Set II, corresponds to a “quasi one-step” batch-foaming. Generally, the term “two-step foaming” is used when samples are first rapidly depressurized without expansion (or a negligible expansion), then expanded out of the vessel by dropping and heating them in an oil or water bath at a chosen temperature (T^foaming^, typically 30 to 100 °C). In two-step foaming, temperature is generally well controlled and constant. In this study, the samples are saturated at different temperatures (from 30 to 100 °C), and pressures (from 7.5 to 31.5 MPa) that leads, or not, to a sample expansion after the pressure drop (at ΔP/dt ~12 MPa·min^−1^). After depressurization the samples are left either at RT or immersed in ice bath (IB) out of the vessel.

### 3.3. Characterization Techniques

Density of materials (unfoamed: ρ_s_ and foams: ρ_f_) were measured using a water pycnometer. Following the water displacement method based on Archimede’s principle, it is possible to determine the density of the material easily. Indeed, for each sample three measurements were done. Then the density was determined with the following Equation (2):

(2) $$\rho =\frac{m_{\mathrm{dry}\;\mathrm{sample}}}{m_{\mathrm{dry}\;\mathrm{sample}}+\Delta m},$$

where m_dry sample_ is the mass of the sample, ∆m is the mass loss between the pycnometer filled only with water and the pycnometer filled with water in which we have added the sample. So, we can write it as in Equation (3):

(3) $$\Delta m=m_{\mathrm{pycno}+H2O}-m_{\mathrm{pycno}+H2O+\mathrm{sample}}.$$

Foams cellular structure was determined on the micrographs obtained with a scanning electron microscopy HITACHI model S-3000N (Tokyo, Japon). The samples were fractured in liquid nitrogen, then gold coated with a sputter coater and observed under a voltage of 10 kV, and a working distance WD = 9 mm, in secondary electron (SE) mode. Determination of the mean cell diameter (ϕ_cell_), cell density (d_cell_), and observations were done with FIJI/ImageJ software (“Image J.2”, 2017).

## 4. Conclusions

Bulk scCO_2_-foamed polymers result from the interplay of three main groups of parameters: (i) Chemical, (ii) physico-chemical, and (iii) technological/process. Polymer solubility in scCO_2_ is often the first order parameter, with the difficulty of the polymer poor solubilities, especially in the short times allowed in continuous processes.

We provided comparative values where literature data have been expressed in the same unit (wt% CO_2_). We summarized the other physico-chemical influencing parameters (e.g., state of the polymer, and Tg).

Then we compared the advantages and drawbacks of continuous (extrusion) continuous (injection) or discontinuous (batch) foaming processes in scCO_2_, especially for micro or nano cellular polymers. Whatever the process, a challenge is to reduce both specific mass (e.g., <0.1·10^3^ kg·m^−3^) and cell size (e.g., average pore diameter ϕ_averg_^pores^ < 100 nm).

Finally, we have presented a particular system where acrylic small “objects” (coreshells CS, or block copolymer MAM) are perfectly dispersed and structured in poly(methyl methcarylate) (PMMA). Some differences between these foaming-aid additives are shown in a one-step batch process. A liquid-core CS presents advantages for a decrease in density, even at room temperature foaming. On another side, in a PMMA/20 wt% MAM blend, through a quasi one-step batch foaming, a “porous to nonporous” transition is observed on thick samples. Such a sharp porosity gradient (from nonporous transparent areas to porous opaque areas within the same sample) would reveal a lower limit of pore size at around 50 nm in a batch classical process in “mild conditions”.

## Figures and Tables

**Table 1:** CO_2_ solubility in polymers: Summary of literature values expressed in % CO_2_ uptake.

Polymers	T (°C)	Pressure (MPa)	CO_2_ Solubility (%)	Reference
**Aliphatic and aromatic hydrocarbons (including polyolefins, polystyrenes and polyethers)**				
hydroxytelechelic	40	25	15	[50]
poly(butylene) (HTPB)				
low density poly(ethylene) (LDPE)	150	0.7–3.5	0.5–2.5	[51]
	25–40	7	0.2	[52]
high density poly(ethylene) (HDPE)	200	6.6–17	3.1–9.3	[53]
	200	10	4.5	[53]
	160	18	13	[53]
poly(ethylene glycol) (PEG)	40	5.3–11.6	11.1–22.6	[54]
400 g/mol	55	3–28	14–30	[55]
1500 g/mol	40	15	23	[56]
non commercial PEG	70	30	50	[57,58]
poly(ether imide) (PEI)	30	0.1	1	[59]
poly(isobutylene) (PIB)	50	20	16	[60]
isotactic poly(propylene) (iPP)	200	6.2–15.4	3–11	[53]
	180	18	14	[53]
	180	11.5	8	[53]
	160	7.5–17.5	5–16	[53]
atactic poly(propylene) (aPP)	120	20	18	[61]
	200	20	14	[61]
atactic poly(styrene) (aPS)	40	30	10	[9]
	80	30	9	[9]
	180	18	7	[53]
	180	10	4	[53]
	100	18.5	11.5	[53]
	180	20	5	[62]
**Carbonyl containing polymers**				
poly(amide) (PA6)	240	5–18	1.2–4	[63]
poly(butylene succinate) (PBS)	120	2.5–20	2–17	[64]
poly(carbonate) (PC)	25	7	13	[65]
Aromatic poly(ether amide) (PEA)	30	0.1	0.9	[59]
poly(ethyl methacrylate) (PEMA)	25	1.4	4.8	[66]
poly(ethylene terephthalate) (PET)	80–120	0–35	0–25	[67]
poly(lactic acid) (PLLA)	40	15	20–25	[68]
poly(methyl methacrylate) (PMMA)	40	10.5	18.2	[69]
	20	30	12.1	[12]
	40	30	16.4	[12]
	100	15	10	[70]
	150	5	3	[70]
	200	20	8	[70]
	50	20	25	[70]
	35	20	30	[70]
	25	7	26	[65]
	25	2	5–7	[71]
	25	1.4	4.4	[66]
	–32	20	48	[42]
poly(vinyl acetate) (PVAC)	25	1.4	6	[66]
**Silicone containing polymers**				
poly(dimethylsiloxane) (PDMS) linear	50	10	25	[69]
	10	20	8.5–10	[69]
crosslinked	35	2	5	[72]
	55	2	4	[72]
	42	20	55	[72]
	42	7	20–30	[72]
**Fluorinated or chlorinated polymers and copolymers**				
poly(vinyl chloride) (PVC)	40–70	5–30	5.5–13	[73]
poly(vinylidene fluoride) (PVDF)	220	10	3	[74]
poly(perfluoro-2-methylene-1,3-dioxolane) (poly(PFMD))	35	1	10	[75]
poly(tetrafluoroethylene) (PTFE)	30	1	2.5	[76]
PS-b-PFDA	0	30	32	[75,77,78]
Other fluorinated copolymers	*	*	*	

* % CO_2_ uptake values are not provided in literature, but these polymers are stated to be very soluble.

**Table 2:** List of influencing parameters on polymer CO_2_ foaming in batch vs. extrusion/injection.

	Batch Foaming	Extrusion Foaming	Injection Foaming
**Process**	Discontinuous	Continuous	Continuous
**Polymer state**	Solid	Initially solid pellets Melted polymer during the process	Initially solid pellets Melted polymer during the process
**CO_2_ role**	Foaming agent	Plastifying effect + foaming agent	Plasticizer (in the extruder) + foaming agent
**Pressure**	Easily controlled into the vessel The depressurization rate can be controlled with a valve.	Indirectly controlled with the screw rate in the barrel, the shearing and with the die geometry Depressurization happens at the end of the die	-Pressure in the injection molding machine as in extrusion foaming Expansion occurs in the mold (mold may be opened at various controlled thicknesses)
**Temperature**	Usually T^foaming^ is close to T_g_ to ensure cell growth during the gas expansion	At the beginning T $\approx T_{melt}$ to melt the pellets Then, depending on the materials’ viscosity the temperature has to be decreased	In the screw zone, same events as extrusion foaming Then, mold temperature is better controlled with heaters or fluid circulation (water or oil)
**Saturation time**	Easy to control Usually long time due to the thickness of the samples	Indirectly controlled by the extrusion rate (linked to the screw rate and the viscosity of the material at the temperature used) Faster than in batch-foaming because polymer is melted	Controlled by the screw speed + the molding time chosen Faster than batch foaming
**References**	[11,87,89]	[86,87,90]	[35,36,91,92]

**Table 3:** Set of experiments I: Characteristics of PMMA-based foams obtained after a one-step batch foaming at a saturation temperature of 80 °C, a saturation pressure of 31.5 MPa and at a gas depressurization speed of ΔP/dt~12 MPa·min^−1^.

PMMA/20 wt% Additive	Average Density (10^3^ kg·m^−3^)	Average Pore Diameter (µm)
MAM	0.29	12.8
D200	0.30	14.5
D480	0.32	13.5
Dlab	0.24	15.5

**Table 4:** Set of experiments II: Density variations observed in various batch foaming conditions (P^sat^, T^sat^).

P^sat^ (MPa)	T^sat^ (°C)	Post Treatment	ρ_pmma/20wt%D200_ (10^3^ kg·m^−3^)	ρ_pmma/20wt%D480_ (10^3^ kg·m^−3^)	ρ_pmma/20wt%Dlab_ (10^3^ kg·m^−3^)	ρ_pmma/20wt%MAM_ (10^3^ kg·m^−3^)
10	30	OS + IB	NF	NF	NF	NF
10 ^a^	30	OS	0.81	0.83	0.7	0.9
10 ^b^	60	OS	0.7	0.71	0.63	-
31.5 ^c^	80	OS	0.3	0.32	0.24	0.29
10 ^d^	80	OS	0.57	0.55	0.56	0.52
7.5 ^e^	100	OS	0.5	0.52	0.5	0.5

OS + IB: One step foaming (OS) followed by an iced water bath (IB) (=quasi one-step foaming). OS: (Classical) one step foaming; *** NF: No foaming, letters (a–f) are used in Figure 3.

**Table 5:** Characteristics of the additives used in a PMMA matrix.

Material	State at T^amb^	Other Characteristics	Density (10^3^ kg·m^−3^)	Aspect
PMMA V825T clear 101	Glassy amorphous solid	Use as polymer matrix	1.19	Transparent
MAM M42	Rubbery center block	Triblock copolymer PMMA-36 wt% PBA *-PMMA	1.18	Transparent
Core shell D ** 200	Crosslinked soft core	PBA core, PMMA shell	1.14	Opalescent
Core shell D480	Crosslinked soft core	PBA core, PMMA shell	1.21	Opaque
Core shell Dlab	Liquid core uncrosslinked	PBA core, PMMA shell	1.13	Opaque

* PBA: Poly(butyl acrylate). ** DuraStrength is a range of commercial core shell particles (CS), with either a crosslinked core or a liquid core, used classically as impact modifiers, MAM is a range of block copolymers (nanostrength), methylmethacrylate-co-butylacrylate-co-methylmethacrylate block- copolymers.
